# Si-Cr Nano-Alloys Fabricated by Direct Femtosecond Laser Writing

**DOI:** 10.3390/ma16051917

**Published:** 2023-02-25

**Authors:** Jovan Maksimovic, Haoran Mu, Molong Han, Daniel Smith, Tomas Katkus, Vijayakumar Anand, Yoshiaki Nishijima, Soon Hock Ng, Saulius Juodkazis

**Affiliations:** 1 Optical Sciences Centre and Australian Research Council (ARC) Industrial Transformation Training Centre in Surface Engineering for Advanced Materials (SEAM), Swinburne University of Technology, Hawthorn, VIC 3122, Australia; 2 Institute of Physics, University of Tartu, W. Ostwaldi Str. 1, 50411 Tartu, Estonia; 3 Optical Sciences Centre, Swinburne University of Technology, Hawthorn, VIC 3122, Australia; 4 Department of Electrical and Computer Engineering, Graduate School of Engineering, Yokohama National University, 79-5 Tokiwadai, Hodogaya-ku, Yokohama 240-8501, Japan; 5 Institute of Advanced Sciences, Yokohama National University, 79-5 Tokiwadai, Hodogaya-ku, Yokohama 240-8501, Japan; 6 Melbourne Centre for Nanofabrication, 151 Wellington Road, Clayton, VIC 3168, Australia; 7 WRH Program International Research Frontiers Initiative (IRFI), Tokyo Institute of Technology, Nagatsuta-cho, Midori-ku, Yokohama 226-8503, Japan

**Keywords:** nano-alloy, Si-Cr, Si nano-needles, sub-100 nm, nanoscale

## Abstract

Ultra-short 230 fs laser pulses of 515 nm wavelength were tightly focused into 700 nm focal spots and utilised in opening ∼400 nm nano-holes in a Cr etch mask that was tens-of-nm thick. The ablation threshold was found to be 2.3 nJ/pulse, double that of plain silicon. Nano-holes irradiated with pulse energies below this threshold produced nano-disks, while higher energies produced nano-rings. Both these structures were not removed by either Cr or Si etch solutions. Subtle sub-1 nJ pulse energy control was harnessed to pattern large surface areas with controlled nano-alloying of Si and Cr. This work demonstrates vacuum-free large area patterning of nanolayers by alloying them at distinct locations with sub-diffraction resolution. Such metal masks with nano-hole opening can be used for formation of random patterns of nano-needles with sub-100 nm separation when applied to dry etching of Si.

## 1. Introduction

Surfaces are fundamentally important for applications in (photo)catalysts, defrosting coatings, anti-bacterial, and biocidal nano-texturing of surfaces, as well as anti-reflection coatings. In most of these cases, the texture is key to the improved performance. However, materials and their composition are another important parameter, which has to be controlled. Surface properties can be also be chemically altered to increase toughness, e.g., Gorilla glass (Corning in 2007) for increased screen durability for mobile phones or handheld computing devices. Another example of tailoring mechanical properties was recently demonstrated in a single-crystal Co${}_{52}$Cr${}_{33}$Al${}_{6.5}$Si${}_{8.5}$ alloy, which was made exhibiting the Young’s modulus of 10–30 GPa similar to that of human bone. It showed high wear and corrosion resistance, as well as super-elasticity with a huge recoverable strain up to 17% [1].

The development of intermetallic compounds and alloys has significantly extended the range of properties of metallic materials [2]. Nowadays, nanoalloys that are fabricated with controllable structures and properties, as well as the flexibility afforded by intermetallic materials, have inspired great scientific and industrial interests and have given rise to diverse applications. These include tailored (photo/electro)-catalyst electrodes used in water-splitting catalysts, green photochemistry, and fertiliser production. In addition, one of the major reasons for interests in nanoalloys is the fact that their chemical and physical properties, especially the catalytic activity [3,4], can be tuned by controlling their surface structures, compositions, and segregation properties [5,6]. For example, nanoalloys, such as AuPd [7], AuIr${}_{2}$ [8], CuPd [3], NiFe [9,10], NiMo [11], RuO${}_{2}$, and IrO${}_{2}$ [12], are efficient electrocatalysts to promote the overall water splitting reactions. These include the hydrogen evolution reaction (HER) [13,14] and oxygen evolution reaction (OER) by facilitating the adsorption/desorption of various ions or regulating the charge transfer [15], owing to the synergistic effects between various intermetallic compounds [16,17,18]. High-entropy alloys (HEAs) [19,20] consist of five or more elements that are considered to be the optimal catalysts due to the millions of different atomic arrangements on their active surfaces, which provides the possibility to overcome the limitations of current catalysts. On the other hand, nanoalloys have also been widely employed in food production applications, and Ag/ZnO alloy nanoparticles have been applied exogenously to wheat plants [21], and Ni-B amorphous alloy catalysts have been utilised to reduce the formation of trans-fatty acids in hydrogenated soybean oil [22], and Co–Mo alloy nanoparticles have been used as highly efficient and stable catalysts for ammonia fertilisers synthesis [23]. Noble metal Au–Ag nano-alloys were shown to perform well for surface-enhanced Raman scattering/spectroscopy (SERS) sensors [24], and Au–Pd is an efficient hydrogen storage material [25]. Families of HEA crystals and metallic glasses with superior mechanical properties are a sharp focus for research and can contribute towards solving the remaining glass transition problem in physics; 2022 is the UN International Year Of Glass.

Laser processing of surfaces affords precise control over the texture and patterning across many types of materials. A technological challenge is large area patterning, with an exemplary case being laser ablated anti-icing micro-patterns on an aircraft wing [26]. The average power of ultra-short sub-1 ps laser pulses has seen exponential growth and fast scanning techniques, such as polygon scanners, which achieve beam travel rates at sonic speeds of hundreds of meters per second. By relying on beam shaping and delivering needle-like tailored Bessel beam or flat-top intensity distributions required for uniform and fast surface processing, the above technological challenges will be solved. Fundamental underlying features of successful three-dimensional laser fabrication/machining/printing relies on the threshold effect, i.e., material undergoes required modification, usually a phase transition, when the correct dose of energy is delivered to the sample/work-piece. Melting, evaporation, and plasma formation at the optical breakdown can be well localised in three-dimensional space. In the same realm of threshold effect, three-dimensional photo-polymerisation takes place when thermal energy can be delivered locally down to nanoscale precision and resolution. Additionally, chemical reactions can be controlled by a strong nonlinear mechanism of light–matter interaction via multi-photon (two-photon is the most efficient among them), avalanche, as well as tunneling ionisation at even higher sides of TW/cm${}^{2}$-to-PW/cm${}^{2}$ intensities per pulse.

Here, different conditions are shown for hard mask patterning by ablation, when nanoscale alloying of the mask material Cr and Si substrate were obtained with high structural control and repeatability. Nano-ring and disk structures made of Si-Cr alloy are demonstrated. Nano-needles with sub 100 nm on Si were observed when dry plasma etching was used. Nano-textures, as well as nano-alloys, can be made using direct laser writing.

## 2. Samples and Methods

Silicon wafers of 〈001〉 surface orientation were used for Cr coating. A subsequent fs-laser exposure was implemented for different types of modification: ablation of a hole in Cr mask for subsequent wet KOH and dry plasma etching of Si, as well as for fabrication of more intricate nanostructures, such as individual nano-rings and nano-disks on the surface of Si.

Chromium was deposited by electron beam evaporation. For its removal, a commercial Cr-etch (Sigma-Aldrich, St. Louis, MO, USA) based on Ceric ammonium nitrate (NH_4_)_2_[Ce(NO_3_)_6_] and perchloric acid HClO_4_ etchant was utilised (∼4 nm/s Cr removal rate when in 85 wt% water solution). SiO_2_ is not affected by the Cr-etch.

Femtosecond (fs-)laser microfabrication setup based on Pharos (Light Conversion) fs-laser was integrated with scanning Aerotech xy-stages and software control of laser emission and scanning conditions (Workshop of Photonics).

Scanning electron microscopy (SEM) was used for structural characterisation of samples processed by laser and plasma treatments (Raith 150TWO electron beam writer was used in field-emission SEM mode). For the elemental analysis, the X-ray energy dispersive spectroscopy (EDS) was carried out with INCA X-act (Oxford instruments Inc., Abingdon, UK) setup on a Supra 40VP (Zeiss, Oberkochen, Germany) SEM instrument.

## 3. Results and Discussion

**Figure 1:**
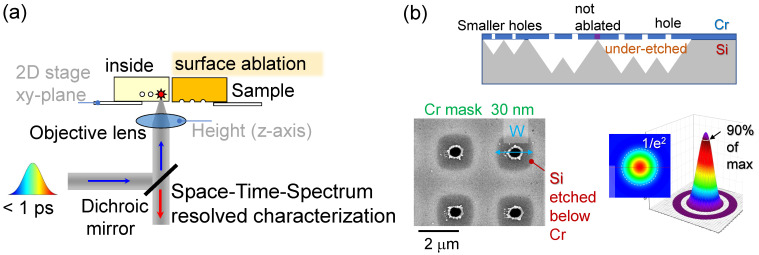
(**a**) Three-dimensional patterning/printing with ultra-short laser pulses with stages delivering lateral xy in-plane positioning and an objective lens height control provides depth (z-axis) position of the focal spot inside of on the surface of the sample. (**b**) Schematics: plasma under-etched profile thorough the holes ablated in Cr mask (20–50 nm thick). SEM image of Cr-mask after etching through ablated holes. Threshold effect of focal intensity distribution tuned to the material modification/ablation level.

Direct fs-laser writing (Figure 1) of etch masks in Cr and Al_2_O_3_ films of tens-of-nanometre thickness was recently demonstrated for photonic crystal (PhC) patterns on a large area (2×2 cm${}^{2}$) for Si solar cell applications [27]. These patterns rely on precisely controlled etching conditions using well defined masks.

### 3.1. Spatially Localised Phase Transitions into Liquid and Plasma States

Parameter study of mask writing by nano-hole ablation shows a different morphology of the ablation area. At pulse energies above ∼10 nJ, irregular ablation craters were formed with molten crown-type splash morphology, while at lower pulse energies, a clear circular rim was formed (Figure 1b). Prolonged ∼15 min plasma etch was not mechanically damaging the mask even when strong lateral under-etch below the mask took place. At low pulse energy ∼1 nJ, Si alloying with Cr was observed as revealed by Cr-etch, which removed all the Cr film not exposed by the laser. Melting temperatures of Cr 1860 ${}^{{}^{\circ}}$C and Si 1414 ${}^{{}^{\circ}}$C are markedly different, which can lead to formation of different alloys. Si-rich phases are expected at lower temperatures closer to the Si-Cr interface: CrSi and CrSi_2_, the latter being a thermoelectric material [28]. Cr-rich alloys are expected at higher temperatures Cr_3_Si, β-Cr_5_Si_3_, and α-Cr_5_Si_3_, which are expected at the centre of the laser pulse exposure site. The thickness of Cr, 20–30 nm, corresponds to the skin depth $\delta_{s}=1/\alpha =4\pi \kappa /\lambda$, hence, is where most laser energy is deposited; the refractive index $\tilde{n}=n+i\kappa$ at λ=515 nm is (2.894+i3.323) and $\alpha =8.108\times 10^{5}$ cm${}^{-1}$ or the skin depth of $\delta_{s}=12.3$ nm (transmittance $T=e^{-\alpha d}\approx 8.8\%$ for d=30 nm of Cr). Strong absorption is defined as αd>1 for a *d* thickness sample. Deposition of laser pulse energy into tens-on-nm depth and localised at the centre of the focal spot delivers high-precision of modification. Formation of Si–Cr alloy competes with oxidation (Cr_2_O_3_ amphoteric, CrO_2_ acidic, CrO basic), which is also kinetically enhanced at high temperatures. A detailed study of surface chemical composition by X-ray photo-electron spectroscopy (XPS) and Raman (for oxides) is required for identification of the compounds formed.

For ablation via ionisation, the electron affinity is important, which is −ΔH=64.3 kJ/mol or −ΔH=6.7666 eV/atom for Chromium, while for Silicon, it is 133.6 kJ/mol or 8.1517 eV/atom. For melting and evaporation, Cr has heat of fusion $H_{f}=20.5$ kJ/mol (394 kJ/kg) and heat of vaporisation 344.3 kJ/mol, while, for Si, it has 50.55 kJ/mol and 384.22 kJ/mol, respectively. This shows that solid–liquid phase transition, as well as ablation (into a plasma state), would be more energy efficient for Cr, rather than Si. Importantly, all the three oxides Cr(II, III, IV) have low melting temperatures in the range 200–300 ${}^{{}^{\circ}}$C, and Cr(VI) CrO_3_ is water soluble (it would not be present on our samples after Cr-etch, which is water-based). It was demonstrated that even low 3 at%. concentration of Si in Cr makes oxidation and volatilization of oxides, as well as nitridation, much slower [29]. Laser ablation is an inherently oxidation-like process since electrons are lost from the surface first after energy deposition by the fs-laser pulse. Better understanding of phase and alloy formation of materials quenched after ultra-short laser exposure is strongly required.

### 3.2. Plasma Etch of Si through an Ablated Mask in Cr Nano-Film

**Figure 2:**
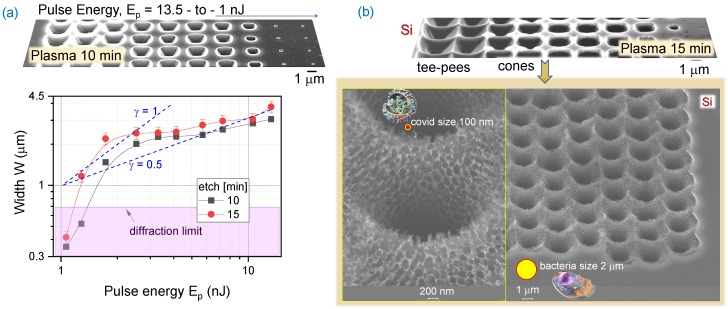
(**a**) The opening width of the etched tee-pee through a hole in 30 nm Cr mask ablated at different pulse energies $E_{p}$. Top-insets shows SEM image after Cr mask removal following a 10 min plasma etch. (**b**) Nano-structures formed below Cr mask in the under-etch region. Etched by SF_6_:CHF_3_:O_2_ at 5:1:1 flow rate ratio.

It was established that the most reliable large-area patterning of Si for PhC light trapping patterns took place when the opening in the 30-nm-thick Cr etch mask was 400–500 nm, i.e., at the sub-diffractional size limit used for focusing into ∼700 nm spot. At this pulse energy range of 2–3 nJ, a ring was clearly discernible and formed at the circumference of the ablated pit. The opening diameter of the hole in the Cr mask affected etch rate (Figure 2a). Additionally, surface morphology of tee-pee etched pits was strongly dependent on the under-etch conditions with adjacent pits merging at the widest opening, right below the Cr mask, as shown in Figure 2a, where coral-like nanoscale black-Si texture was formed. Under prolonged etching (∼10 min), when deeper structures were formed, surface morphology became nano-smooth.

**Figure 3:**
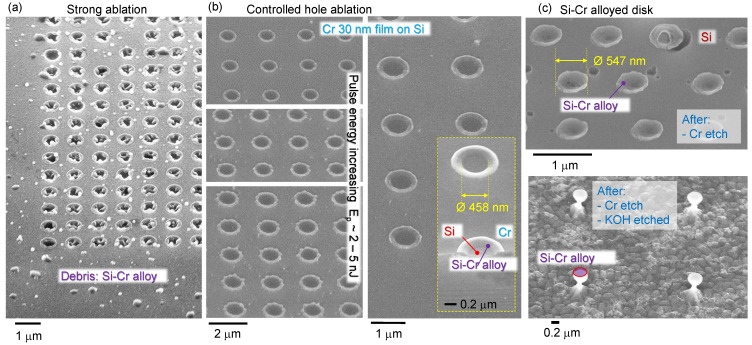
Pulse energy control: hole, ring, disk, nanoparticles. (**a**) SEM image of Cr 30 nm mask ablated by 515 nm/230 fs single pulses focused with NA=0.9 objective lens; pulse energy $E_{p}>12$ nJ (on sample). (**b**) Controlled opening of mask window at 2–5 nJ pulse energies. Inset shows tilted view of cross section. (**c**) Focal center made from alloyed Si and Cr with sub-diffractional diameter <700 nm disks. Pulse energy $E_{p}=1$ nJ.

**Figure 4:**
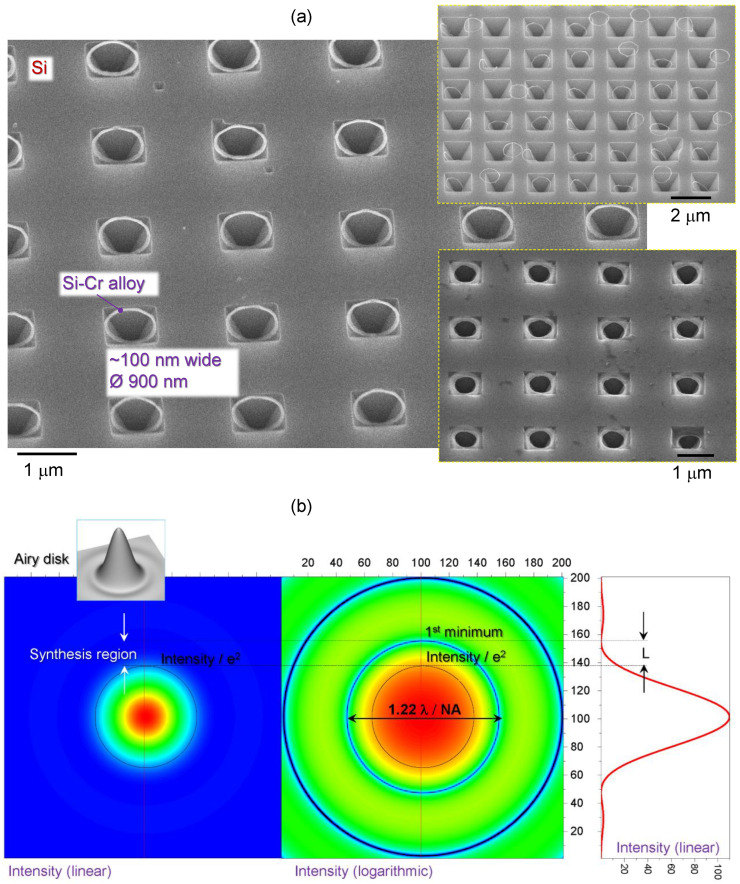
(**a**) SEM image of Si-Cr alloy ring formed at the rim of ablated pit in the Cr mask; imaged after KOH etch of Si (inverted pyramids are formed) and Cr-etch removal of the laser unexposed regions of Cr. Insets shows conditions of KOH over-etch when opening of the pyramidal pit becomes larger as diameter of the ring. (**b**) Intensity distribution of a plane wave at the focus in linear and logarithmic scale; the inset shows a 3D envelope of the intensity profile (grey).

When pulse energy was ∼1 nJ/pulse, the surface of Cr was distinctively changed, however, there was no hole opening. A Si–Cr alloy was formed on those islands ∼500 ± 50 nm. Figure 3 shows patterns fabricated by 1–10 nJ pulses ranging from strong ablation of holes and ablation debris fields to controlled melting and alloying nano-film of Cr (30 nm) with underlying Si; see Appendix A for energy dependence of hole formation on a 10-nm-thick Cr on Si. Those alloyed Si–Cr nano-disks were not affected by Cr-etch, which confirms their altered composition. Similarly, the rings formed around ablated holes at larger pulse energies were also not dissolved in Cr-etch, as clearly illustrated by the rings defining the rim of the ablated hole in the Cr mask after KOH etched inverted pyramidal pits in Si (Figure 4a). During removal of Cr from the substrate, it is submerged in a liquid etchant. In this environment, rings/discs can dislodge from the pyramids, and is most relevant when the inverted pyramid is over-etched to a size larger than the rings. This leads to the disordered positioning of the rings in the Figure 4a inset.

Figure 4b shows focal intensity distribution (normalised) of a plane wave given by the Airy function $I\propto {\left(2J_{1}\left(x\right)/x\right)}^{2}$, where $J_{1}\left(x\right)$ is the Bessel function of the first kind of order one, x=kasinθ with k=2π/λ as the wavevector, *a* being the radius of aperture, and θ an observation angle (from the aperture). The rings of ∼100 nm width were located close to the first minimum of the Airy disk on the focal spot.

### 3.3. Nano-Disk Alloy of Si-Cr

Ultra-short pulses deliver high intensity but low pulse energy, favouring precise energy deposition. Small volumes at the focal spot can be heated and ablated by deposited energy. Thermal quenching of the small volume can also facilitate precision and localisation of laser induced modification. This reduces surface tension σ-driven flows. For example, molten Cr has surface tension of σ=1.72 N/m at T=2148 K [30] and was found following linear scaling with temperature. Chromium alloy with Ni was found following Bulter’s equation $\sigma =\frac{\mu_{S}-\mu_{B}}{a}$ of individual metals according to the mixing volume ratios, where $\mu_{S,B}$ are chemical potentials of surface and bulk, respectively, and the number of atoms per surface area $a=1.09\sqrt[{3}]{V^{2}}\sqrt[{3}]{N_{A}}$ with $N_{A}$ is the Avogadro number, and *V* is the molar volume of pure metal.

**Figure 5:**
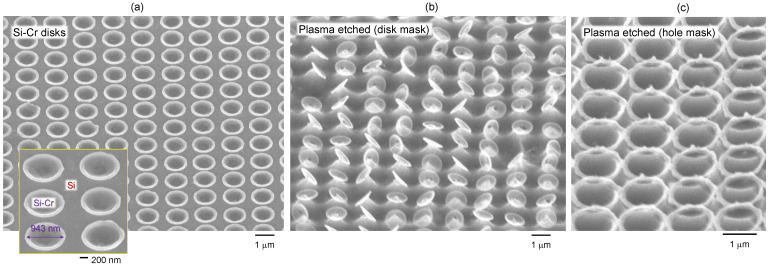
(**a**) SEM image of the Si–Cr alloy disk formed on the Cr film (30 nm), imaged after Cr-etch. Pulse energy $E_{p}=3$ nJ. (**b**) SEM images of Si-Cr disks used as dry plasma etch mask followed by Cr-etch. (**c**) SEM image of Si-Cr rings used as dry plasma etch mask followed by Cr-etch. Laser patterning was carried out with NA=0.75; diffraction limited focal diameter 1.22λ/NA=838 nm; wavelength λ=515 nm. Plasma etch was for 5 min.

Figure 5a shows an array of Si–Cr alloyed disks made by single pulse exposure with an NA=0.75 objective lens (Nikon). Those disks were used as a mask during dry plasma etching and were imaged after Cr-etch removed the remaining pristine Cr mask (Figure 5b). Nano-disks were affected by capillary forces when taken out of the aqueous-based Cr-etch. This caused disk pattern distortion, however, it is obvious that nano-disks of ∼30 nm thickness provided a mask function during the 5 min plasma etch. Similarly, ablated holes in Cr mask were used for plasma etch, followed by Cr removal (Figure 5c). This shows that plasma etch, as well as KOH wet etch (Figure 4a), can be used for etching through ablated holes with Si-Cr alloyed rings at the circumference.

Let us estimate energy deposition in to 30 nm Cr film on Si. For $E_{p}=1$ nJ, λ=515 nm and $t_{p}=230$ fs pulses focused onto 2r=1.22λ/NA=700 nm (NA=0.9), the fluence per pulse is $F_{p}=0.26$ J/cm${}^{2}$, average intensity $I_{p}=F_{p}/t_{p}=1.14$ TW/cm${}^{2}$ (the peak intensity is $2I_{p}$ assuming Gaussian pulse).

Specific heat capacity of Cr is $c_{s}=460.55$ J/(kg.K) [Si: 711.76 J/(kg.K)], hence the energy required to heat up cylinder volume $V_{f}$ of d=30 nm Cr and 2r=700 nm diameter to the melting temperature of $T=1860{\;}^{\circ }$C (from RT 20 ${}^{\circ }$C) is $Q_{T}=c_{s}\varrho V_{f}\Delta T=80$ pJ where mass density of Cr is ϱ=7150 kg/m${}^{3}$. To melt that volume, it takes $Q_{m}=H_{f}\varrho V_{f}=32.4$ pJ, where $H_{f}=394$ kJ/kg [Si: 1787 kJ/kg]. Total energy $\left(Q_{T}+Q_{m}\right)$ required to melt Cr layer of 30 nm over the focal diameter takes only 11.2% of the incident 1 nJ pulse. The same estimate for 30 nm thick Si is $Q_{T}+Q_{m}=31.8+47.8$ pJ, where mass density ϱ=2330 kg/m${}^{3}$ or ∼8% from the 1 nJ pulse. Even considering that portion of incident 1 nJ pulse is reflected and scattered, which can be estimated from *n* and κ, there is an energy budget for surface melting and further heating of the molten phase. The estimates here are qualitative only since they do not account for the nonlinear light–matter interaction and ablation. Typical ablation threshold for metals under sub-1 ps pulse exposure is ∼0.1–0.2 J/cm${}^{2}$, and it corresponds closely to the 1 nJ pulse energy used in this estimate and in experiment. Previously, controlled oxidation of Si under high repetition rate of fs-laser irradiation was used for inscription of SiO_2_ plasma etch grey-scale masks at such sub-ablation threshold conditions [31]. Thermal control of melting and re-solidification for amorphous-crystalline transitions can be more deeply explored for high spatial resolution patterning, especially using burst mode of fs-lasers.

**Figure 6:**
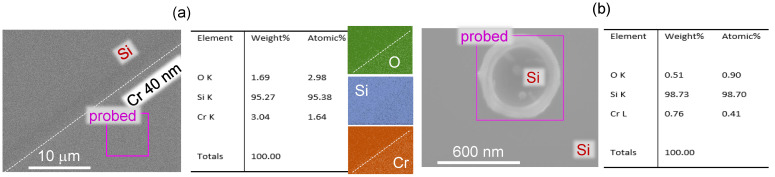
X-ray energy-dispersive spectroscopy (EDS) area scan for chemical composition of O, Si, Cr. (**a**) A film of 40 nm Cr was coated on Si substrate before laser exposure. Edge between Si and Cr coating was determined by a cover glass rim during e-beam evaporation. (**b**) A Cr–Si alloyed ring at pulse energy of $E_{p}=1.1\pm 0.1$ nJ (on sample); λ=515 nm wavelength, objective lens of NA=0.9. Cr was washed out after the laser exposure by Cr-etch; surface around the ring is Si.

Figure 6 shows X-ray energy dispersive spectroscopy (EDS; INCA X-act, Oxford instruments Inc. setup on Supra 40VP, Zeiss SEM) results for elemental change induced by single fs-laser pulse. Since EDS collects compositional information from the deep sub-surface (micrometre) region, we compared the change of Cr-to-O ratio before and after irradiation of 40 nm thick Cr coating. Cr coating of a 40 nm thickness showed $\frac{O}{Cr}=1.82$ at% (reference) and changed to $\frac{O}{Cr}=2.20$ at% at the irradiated site after Cr etch. Only 3.6% change was observed in change of Si before and after irradiation, which was the main contribution to EDS signal from the sub-surface. More Si was in the laser irradiated and Cr-washed case. It should be noted that with the large interaction volume and, hence, low resolution of the technique, these values still only indicate a qualitative change in composition. It is apparent that the Cr nanofilm was partially oxidised at the start, and (Cr forms a passivisation layer when exposed to air), however, such film was fully washable by Cr-etch. After laser irradiation and ablation, the rim of Si-Cr alloy was only slightly smaller than the focal spot size of 1.22λ/NA≈700 nm and was robust against wet etch of Cr. Alloying of Si and Cr and oxidation needs further dedicated studies with nanoscale resolution. Techniques, such as electron energy loss spectroscopy and EDS with transmission electron microscopy (EELS), can fulfil those needs.

### 3.4. Optical Properties of Fabricated Patterns

**Figure 7:**
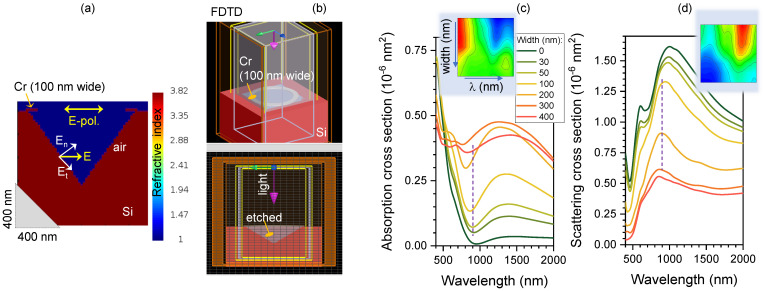
FDTD simulations (Lumerical ANSYS). (**a**) Refractive index cross section of a 30-nm-thick and 100-nm-wide Cr ring with outer diameter 950 nm. The linear polarisation of E-field is projected at the Si–air interface as normal and tangentical components ($E_{n},E_{t}$). (**b**) Calculation volume in slanted and side views. (**c**,**d**) Absorption and scattering cross sections $\sigma_{ab,sc}$, respectively, for the different width Cr rings with outer diameter 950 nm and 30 nm thickness. Inset pictograms show map diagrams with blue-red color scale corresponding low-high $\sigma_{ab}$ (**c**) and $\sigma_{sc}$ (**d**). Material properties are taken from Lumerical database, which refers to Palik’s data set.

A metal/alloy nano-ring positioned above etched inverted pyramid in Si surface can be made on large macroscopic areas with cross sections >1 mm. Optical response of such patterns under normal incidence was simulated with finite difference time domain (FDTD, Lumerical, ANSYS) method at different wavelengths, which are larger and smaller than the opening of the nano-ring (Figure 7a,b). In order to model the Si–Cr alloy, it is necessary to have its refractive index over the range of wavelengths. It was not possible to measure this, so Cr and Au were used as model metals; see Appendix B. Absorption and scattering cross sections $\sigma_{ab}$, $\sigma_{sc}$ were calculated for different widths of the Cr-ring for the fixed diameter 950 nm; the extinction cross section is $\sigma_{ex}=\sigma_{ab}+\sigma_{sc}$ (Figure 7c). Extinction is dominated by scattering for rings with small widths while absorption losses become larger for wider rings; thickness of Cr was 30 nm, as in experiments.

**Figure 8:**
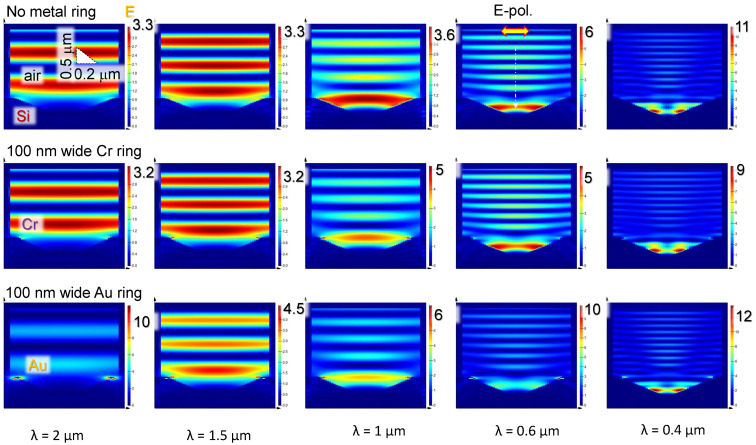
FDTD simulations (Lumerical ANSYS). Reflection from a structure of 100-nm-wide (30-nm-thick) Cr or Au ring of 900 nm diameter on an etched inverted pyramid on Si 〈001〉 plane at different wavelengths; top-row is reference with no metal ring. Color map shows light enhancement; incident light is E=1. Calculations were carried out with periodic boundary conditions (in surface plane of Si wafer). Material properties are taken from Lumerical database. Note the different dimensions in horizontal and vertical directions.

Chromium and gold (perfect metal) were used for comparison of light localisation and enhancement (Figure 8). Obviously, Au caused strong E-field enhancement, and its localisation was at the Si–Au boundary. The chromium nano-ring caused minor effects of light localisation in its vicinity, however, the overall light intensity distribution was not markedly different from situation when it was absent (top row in Figure 8). For shorter wavelengths, formation of intensity maxima at the edge of inverted-pyramid boundary with air becomes obvious. This is a consequence of the displacement’s εE normal to the interface component being continuous in Si and air, where the interface has no conductivity, and, here, $\varepsilon \equiv {\tilde{n}}^{2}$ is permitivity. This causes light enhancement $\varepsilon_{Si}E_{n}^{Si}=1\times E_{n}^{air}$, i.e., intensity in air is $I\propto E_{air}^{2}$, which is $\varepsilon_{Si}^{2}=n_{Si}^{4}\approx 150$ times larger than inside Si for $n_{Si}=3.5$. This enhancement is only for the normal component to the interface with the 〈111〉 side plane of the inverted pyramid $E_{n}=\cos\left(\pi /2-\beta \right)E_{inc}$, where $\beta =54.7^{\circ }$ is the angle between the top surface (001) and side wall of pyramid (111) and $E_{inc}$ is linearly polarised (horizontal in Figure 8) incident E-field. Such enhancement can be used for nano-ablation by near-field in nano-grooves [32]. E-field perpendicular to the interface is useful for photo-catalytic applications [33]. For surface textures used for light trapping in Si solar cells, such enhancement at the interface promotes absorption, however, surface recombination rate on a large and modified surface is also enhanced. In the case of amorphous-Si, the minority carrier lifetime $\tau_{mc}$ measured by photo-conductance (Sinton) is typically 40–90 μs for the range of $10^{15}$–$10^{17}$ cm${}^{-3}$ p-type doping in the case of non-passivated surfaces [34]. Hydrogenation and/or thermal annealing is used to increase minor carriers recombination lifetime to few-ms range. This is of paramount importance for thin Si solar cells since $\tau_{mc}\propto W/\left(2S\right)$, where *W* is thickness of solar cell, and *S* [cm/s] is the surface recombination velocity.

The same boundary condition discussed above is applicable to the conductive ring and defines the local normal E-field according to the condition $\varepsilon_{Si}E_{n}^{Si}-1\times E_{n}^{air}=\rho_{Cr}$, where $\rho_{Cr}$ is surface charge density of metallic ring of Cr (here for air $\varepsilon \equiv n^{2}=1$). When electrically conductive materials/structures are on the surface of a photo-electrode, the local E-field enhancement can take place for the normal component.

## 4. Conclusions and Outlook

It is shown that alloying a nano-film with a substrate (underneath it) can be achieved with precise control of energy deposition during ablation, when nano-ring structures are formed, and a controlled remelting forms nano-disks below the ablation threshold. This adds another dimension to laser nano patterning and complements physical nano-texturing. Nano HEA alloys have previous been produced using laser-synthesis as nanoparticles [35]. At the threshold of ablation of a metal ∼0.2 J/cm${}^{2}$/pulse corresponds to ∼1 nJ/pulse at tight focusing with high-NA = 0.9 and 0.75 dry objective lens. Fine control over the energy delivered allows the alloy to remain on the substrate, allowing more flexible patterning compared to nanoparticle generation. Controlled melting and alloying of surface deposited nano-films by ultra-short pulse direct writing is a promising technique for (photo-)catalytic applications. Selective alloying and nanoscale materials processing opens up bespoke control over alloy composition and precise localisation on the 100 nm scale. Due to a small volume, with lateral cross sections ∼200–400 nm and ten times smaller depth, thermal quenching of the liquid phase is fast and can capture non-equilibrium phases of alloys at different degrees of mixing. Applications in metallic glasses and high entropy alloys (HEA) are the most obvious beneficiaries of the proposed method. Direct write of Cr nano-disks shown here can be used for lithography-free direct patterning of surfaces of MIM metasurfaces where Cr is required to increase absorption of the top metallic nano-disks [36]. It is shown here that pulse energy deposition has to be controlled with ∼0.1 nJ/pulse for phase transitions and alloying for sub-wavelength structures/patterns.

## Figures and Tables

**Figure A1:**
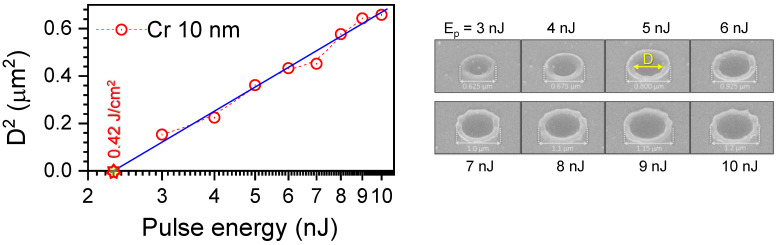
Central hole diameter *D* opened in a 10-nm-thick Cr film on Si under one pulse irradiation at different pulse energies $E_{p}$ (on the sample). Wavelength of laser pulse was 515 nm, pulse durartion 230 fs, focused by NA=0.75 numerical aperture objective lens. Slanted-view SEM images show evolution of hole opening (diameter *D*) at different pulse energies $E_{p}$.

**Figure A2:**
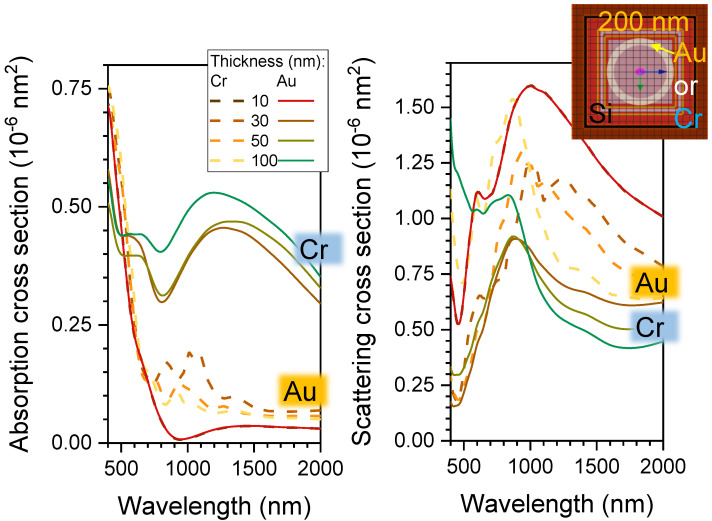
FDTD simulations (Lumerical ANSYS). Cross sections $\sigma_{ab}$ and $\sigma_{sc}$ of 200-nm-wide Cr or Au (dashed-lines) rings of 950 nm diameter and different thicknesses from d=10 to 100 nm. Material properties are taken from Lumerical database.

## Data Availability

Data available on request.

## Appendix A. Ablation of a Hole in Cr Nanofilm on Si

The threshold of hole formation on a 10-nm-thick Cr film on Si was measured using standard $D^{2}$ vs. $\mathrm{lg}(E_{p})$ plot (Figure A1). The inner diameter was measured. The rim of Cr-melt and possible Si-Cr alloy was 150±20 nm. The threshold was found at $E_{p}=2.3$ nJ/pulse (on the sample) or fluence of $F_{p}=0.42$ J/cm${}^{2}$ corresponding to irradiance of $I_{p}=F_{p}/\tau_{p}=1.8$ TW/cm${}^{2}$ for $\tau_{p}=230$ fs pulse duration. The fluence is approximately double than would be expected for Si. This can be caused by a larger melting temperature of Cr as compared with Si. Melting of Cr (rather ablation) was obvious at the used small pulse energies. The actual size of hole opening *D* is affected not only by the direct ablation, but also by hydrodynamics of a molten phase driven by strong temperature gradients, hence, surface tension gradients. A colder region of molten material is pulling the hotter liquid towards the rim of the opening.

## Appendix B. FDTD Modeling of Optical Properties

Figure A2 Shows dependence of scattering and absorption cross sections on the thickness of ring simulated for Cr and Au. Au had about four-times lower absorption as compared with Cr and an opposite trend for scattering. This is consistent with a perfect absorber-emitter study of metal-insulator-metal (MIM) metasurfaces where a thicker Cr layer of 50 nm had to be used together with Au nano-disks to increase the cumulative absorbance of the MIM structure [37].
